# CT-like MR-derived Images for the Assessment of Craniosynostosis and other Pathologies of the Pediatric Skull

**DOI:** 10.1007/s00062-022-01182-x

**Published:** 2022-06-28

**Authors:** Yannik Leonhardt, Sophia Kronthaler, Georg Feuerriegel, Dimitrios C. Karampinos, Benedikt J. Schwaiger, Daniela Pfeiffer, Marcus R. Makowski, Inga K. Koerte, Thomas Liebig, Klaus Woertler, Marc-Matthias Steinborn, Alexandra S. Gersing

**Affiliations:** 1 Department of Radiology, Klinikum Rechts der Isar, School of Medicine, Technical University of Munich, Ismaninger Straße 22, 81675 Munich, Germany; 2 Department on Neuroradiology, Klinikum Rechts der Isar, School of Medicine, Technical University of Munich, Munich, Germany; 3 Psychiatric Neuroimaging Laboratory, Brigham and Women’s Hospital, Harvard Medical School, Boston, MA USA; 4 Department of Child and Adolescent Psychiatry, Psychosomatic, and Psychotherapy, Ludwig-Maximilians-Universität, Munich, Germany; 5 Department of Neuroradiology, University Hospital of Munich (LMU), Munich, Germany; 6 Department of Diagnostic and Interventional Radiology and Pediatric Radiology, München Klinik Schwabing, Munich, Germany

**Keywords:** Magnetic resonance imaging, Skull, Cranial bone imaging, Children, Radiation exposure

## Abstract

**Purpose:**

To evaluate the diagnostic value of CT-like images based on a 3D T1-weighted spoiled gradient echo-based sequence (T1SGRE) for the visualization of the pediatric skull and the identification of pathologies, such as craniosynostosis or fractures.

**Methods:**

In this prospective study, 20 patients with suspected craniosynostosis (mean age 1.26 ± 1.38 years, 10 females) underwent MR imaging including the T1SGRE sequence and 2 more patients were included who presented with skull fractures (0.5 and 6.3 years, both male). Additionally, the skull of all patients was assessed using radiography or CT in combination with ultrasound. Two radiologists, blinded to the clinical information, evaluated the CT-like images. The results were compared to the diagnosis derived from the other imaging modalities and intraoperative findings. Intrarater and interrater agreement was calculated using Cohen’s κ.

**Results:**

Of the 22 patients 8 had a metopic, 4 a coronal and 2 a sagittal craniosynostosis and 2 patients showed a complex combination of craniosynostoses. The agreement between the diagnosis based on the T1SGRE and the final diagnosis was substantial (Cohen’s κ = 0.92, 95% confidence interval (CI) 0.77–1.00 for radiologist 1 and κ = 0.76, CI 0.51–1.00 for radiologist 2). Of the patients with fractures, one presented with a ping pong fracture and one with a fracture of the temporal bone. Both radiologists could identify the fractures using the T1SGRE.

**Conclusion:**

The visualization of the pediatric skull and the assessment of sutures using a CT-like T1SGRE MR-sequence is feasible and comparable to other imaging modalities, and thus may help to reduce radiation exposure in pediatric patients. The technique may also be a promising imaging tool for other pathologies, such as fractures.

## Introduction

Craniosynostosis is the premature closure of one or more of the cranial sutures [1]. Although relatively rare with an incidence of approximately 2.6–6.4 in 10,000 children [2, 3], craniosynostosis is a clinically relevant condition. As the skull cannot grow adequately in the affected areas, the shape of the head is often distorted, and the disparity between the growing brain and the restricted growth of the skull can lead to raised intracranial pressure. Therefore, an early surgical correction is often necessary [4]. Diagnostic imaging is usually necessary in order to confirm the diagnosis and for preoperative planning [5]. Moreover, there are other pathologies, such as skull fractures, in which imaging is necessary in order to plan the following therapeutic steps, which may even imply surgery. Radiography or, more commonly, low-dose computed tomography (CT) is often performed, since especially CT enables a highly detailed visualization of sutures, bone structure and skull shape [5–7]; however, radiation exposure from those modalities is of concern in pediatric patients [8, 9]; therefore, alternatives should be explored.

Magnetic resonance (MR) imaging is primarily used for the examination of the intracranial structures; however, visualization of the skull using MRI as a radiation-free method has been investigated in the past. In 2012 Eley et al. introduced a gradient echo sequence with short echo time (TE), short repetition time (TR) and a flip angle of 5° for imaging of the head and neck region, which produces images with low signal in osseous structures and results in a high contrast to the surrounding soft tissue. Due to the hypointense or black appearance of the osseous bone components, imaging using this sequence and similar variants is referred to as black bone (BB) imaging. In subsequent studies Eley et al. applied the sequence to 17 pediatric patients and showed that BB-MR imaging allows 3D visualization of the skull, sutures, and craniofacial skeleton [10–12]. In 2018, Kuusela et al. used the variant of BB imaging derived from a T1 VIBE sequence, which was acquired using two echo times (in phase and out of phase), and applied this sequence to a study group of 15 pediatric patients [13]. Moreover, a fast low-angle shot golden-angle 3D stack-of-stars radial VIBE- (GA-VIBE-) based sequence on a 3 T MRI, with similar TR/TE and a flip angle of 3° was applied and evaluated in 11 pediatric patients [14]; however, 3D reconstruction and post-processing of the BB sequences are fairly extensive and time consuming, which still limits the application in the clinical routine. This problem was approached by automated segmentation techniques; however, these techniques were applied to BB-MR images of the skull of adults. The application of these techniques in pediatric patients might be challenging due to the different composition of the osseous, cartilaginous and soft tissue structures [15, 16].

In other studies that explored bone imaging using MR, a 3D T1w spoiled gradient-echo-based sequence at single echo time (3D T1SGRE) was investigated for the visualization as well as the assessment of bone structures and morphology. This sequence generates high-resolution MR images that “simulate” detailed, CT-like images after inversion [17]. This sequence has proved to allow the diagnosis of vertebral fractures and degenerative changes of vertebrae and subtle findings of bone tumors with a high diagnostic accuracy and agreement with CT and radiographic images [17, 18].

The purpose of this study was therefore to evaluate the diagnostic value of CT-like images based on the 3D T1SGRE technique for the radiation-free visualization of the pediatric skull and the identification of pathologies, such as prematurely fused cranial sutures or fractures.

## Methods

### Patient Selection

This prospective study was approved by the local institutional review board and informed consent was obtained prior to the participation in the study from the legal guardian of the participants, since the age of all participants was below 18 years. From August 2021 until February 2022 patients with suspected skull pathologies who were scheduled for a cranial MR examination at our institution as part of the clinical routine were approached for this study.

**Fig. 1:**
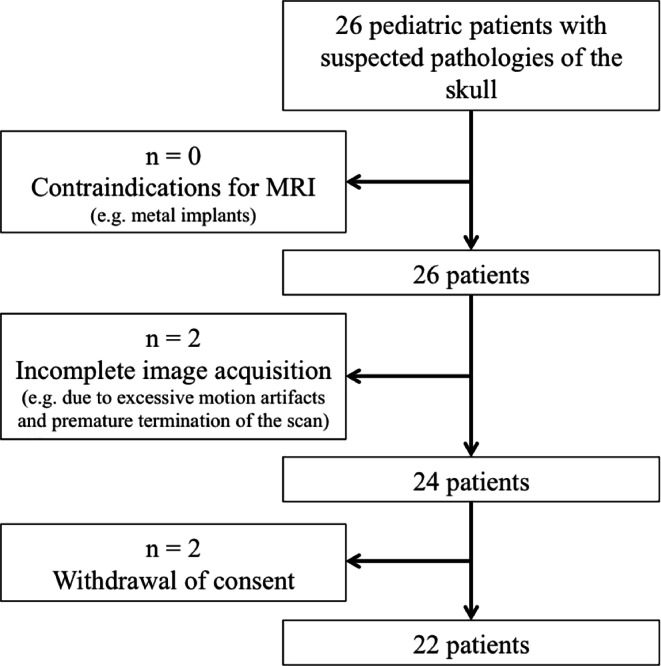
Flowchart of patient selection

In total, 22 patients with suspected pathologies of the pediatric skull (mean age 1.29 ± 1.40 years; 10 females) were included in our study. Exclusion criteria were general contraindications for MR imaging (e.g. pacemaker or other metal implants; *n* = 0), incomplete image acquisition (e.g. due to excessive motion artifacts and premature termination of the scan; *n* = 2) and withdrawal of consent (*n* = 2) (Fig. 1). In this study population, the T1SGRE sequence was acquired in the same imaging session as the MR examination that was performed as part of the clinical routine diagnostic work-up. All patients were examined in general anesthesia. Within 3 months before or after MR imaging, further imaging modalities, such as radiography or CT and ultrasound of the skull were performed. Surgery was performed in 15 of the 20 patients with suspected craniosynostosis.

### MR Imaging and Post-processing

MR images were acquired using a 3T MR scanner (Philips Achieva; Philips Healthcare, Best, The Netherlands) as well as using a 32-channel head coil. A 3D T1-weighted spoiled gradient echo-based sequence (T1SGRE) was acquired with the following parameters: in-plane spatial resolution 0.3 mm × 0.3 mm; TR 10 ms; TE 2.1 ms; flip angle 8°; slice thickness 0.5 mm; field of view (FOV) 200 × 200 × 200 mm^3^; average scan time 4 min 55 s. Partial Fourier imaging was applied in the frequency encoding direction (60%) to minimize the echo time. Further clinical sequences were also obtained in this session (FLAIR, T2, T1, DWI, ADC); the average overall scan time including the 3D T1SGRE sequence was 21 min 6 s. For the evaluation of osseous structures, the 3D T1SGRE sequence was reformatted in sagittal, coronal, and axial orientation with a slice thickness of 1 mm, gray scales were inverted, and windowing was set to resemble a CT bone window. Moreover, 3D reformations of the skull surface were generated using the open-source software platform 3D Slicer (version 4.10.2; https://www.slicer.org/).

### CT

All CT examinations were performed on one CT scanner (Somatom Sensation 40, Siemens Healthineers). The data were acquired in helical mode with a peak tube voltage of 120kVp, a slice thickness of 0.75 mm, and modulated tube current of 200 mAs. Images were reformatted in 1 and 5 mm slice thicknesses using a bone-specific convolution kernel.

### Image Analysis

The images were individually and independently read by two radiologists ASG (radiologist 1) with 10 years and YL (radiologist 2) with 3 years experience in radiology, blinded to clinical information and all other patient information including all MR sequences other than the T1SGRE. The radiologists read the images in a random order and recorded the findings blinded from each another. The degree of fusion of each suture (sagittal, right and left coronal, right and left lambdoid, metopic) was graded (0, open; 1, partially fused; 2, completely fused). If present, the main localization of the craniosynostosis was assessed. The deformity of the skull was classified as normocephaly, scaphocephaly, trigonocephaly, brachycephaly or plagiocephaly using the 3D reconstructions. Presence and location of a potential fracture were assessed. Furthermore, MR images were graded for sharpness, noise and overall image quality on a 4-point Likert scale (score of 1, excellent; 2, good; 3, moderate; 4, poor). Certainty of diagnosis was evaluated in 6 grades (0, 20, 40, 60, 80 and 100%). The final diagnosis, made intraoperatively in combination with information of other imaging modalities, such as radiography or CT and ultrasound of the skull, was used as standard of reference. Image interpretation of the radiographs or CT and ultrasound of the skull was performed by a pediatric radiologist (MMS, 20 years experience in pediatric radiology).

### Statistical Analysis

The data were analyzed using IBM SPSS Statistics for Windows, version 27.0 (IBM Corp., Armonk, NY, USA). All statistical tests were performed two sided, and a level of significance (α) of 0.05 was assumed for all tests. The agreement between the diagnosis based on MR-derived CT-like images and the final diagnosis was calculated using Cohen’s kappa. Sensitivity and specificity were calculated for each reader. The interobserver and intraobserver reliability of the assessment of the MR-derived CT-like images was calculated using Cohen’s kappa. Intraobserver reliability was based on a second evaluation of all image studies by both radiologists 4 weeks after the first assessments. The values can be interpreted as poor (0), slight (0.0–0.2), fair (0.21–0.40), moderate (0.41–0.60), substantial (0.61–0.80) and almost perfect (0.81–1.00). For all measures the 95% confidence intervals (CI) were calculated.

## Results

In total, 22 patients with suspected pathologies of the pediatric skull (mean age 1.29 ± 1.40 years; 10 females) were included in our study. Of the 22 patients, 20 presented with suspected craniosynostosis (mean age 1.26 ± 1.38 years, 10 females) and 2 with fractures of the skull (mean age 3.40 ± 2.90 years, both male) (Table 1).

**Table 1:** Patient characteristics

	Total (*n* = 22)	Craniosynostosis (*n* = 20)	Fracture (*n* = 2)
*Age (years)*	1.29 ± 1.40	1.26 ± 1.38	3.40 ± 2.90
*Sex (n)*			
Male	12	10	2
Female	10	10	0

### Patients with Craniosynostosis

Of the 20 patients with suspected craniosynostosis 8 had metopic, 4 coronal and 2 sagittal synostosis as final diagnosis. Two patients presented with a complex combination of craniosynostoses as manifestation of the Crouzon syndrome. The remaining 4 patients showed no craniosynostosis. Corresponding to the above-described synostoses 8 patients presented with trigonocephalus, 4 with brachycephalus and 2 with scaphocephalus. The two patients with Crouzon syndrome showed complex skull deformities. The frequencies of each pathology assessed are listed in Table 2.

**Table 2:** Frequencies, *n* (%) of evaluated criteria, as rated by radiologist 1

Characteristic	Frequency, *n* (%)
*Primarily affected suture*	
Sagittal	2 (9.1)
Right coronal	1 (4.5)
Left coronal	2 (9.1)
Right lambdoid	0 (0)
Left lambdoid	0 (0)
Metopic	8 (36.4)
*Deformity of the skull*	
Normocephaly	6 (27.3)
Scaphocephaly	2 (9.1)
Trigonocephaly	8 (36.4)
Brachycephaly	3 (13.6)
Plagiocephaly	0 (0)
*Degree of fusion of the main suture*	
Full	7 (53.8)
Partial	6 (46.2)
*Fracture present*	
Yes	2 (9.1)
No	20 (90.9)

**Fig. 2:**
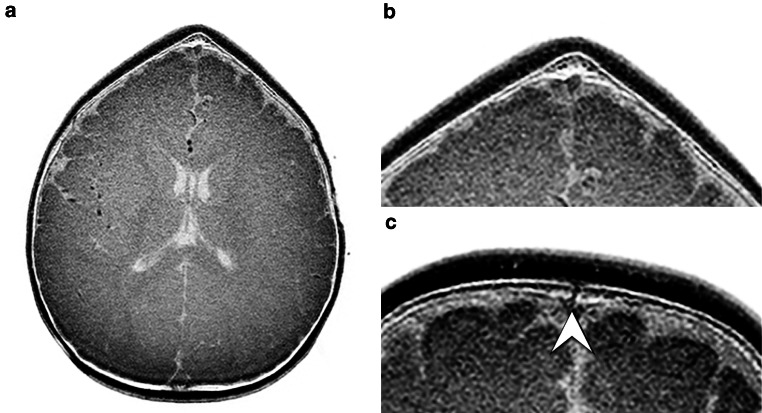
Inverted axial reformation of the 3D T1-weighted gradient echo sequence of the skull of a 5-month-old male patient with metopic synostosis and trigonocephalus (**a**), showing the fused metopic suture of this patient in an axial plane (**b**). Inverted axial reformation of the 3D T1-gradient echo sequence of the skull of a 6-month-old male patient with a patent metopic suture (**c**), indicated by the arrowhead. It should be noted that the lamina interna, diploe and lamina externa can clearly be differentiated from each other on these images

**Fig. 3:**
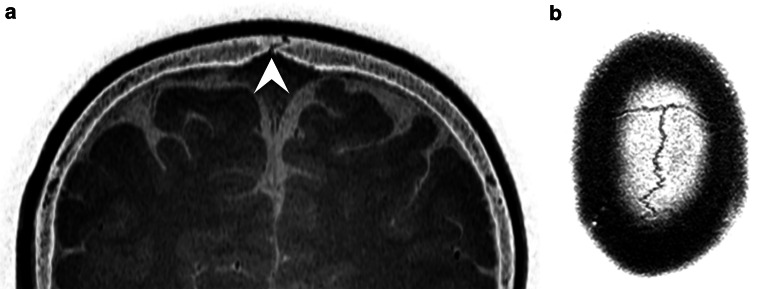
Inverted coronal and axial reformations of the 3D T1-gradient echo sequence of the skull of a 6-year-old male patient without craniosynostosis (**a** and **b**). Details like the sutures (as indicated by the arrowhead) can clearly be depicted on these images

Diagnosis based on the 2D CT-like images of the patients with suspected craniosynostosis (Figs. 2 and 3) showed a substantial to almost perfect agreement with the final diagnosis for both radiologists (Cohen’s κ coefficient = 0.92, 95% CI, 0.77–1.00 for radiologist 1 and κ = 0.76, 95% CI, 0.51–1.00 for radiologist 2) (Table 3). Assessment of skull deformities based on the 3D reconstructions of the CT-like images showed a substantial to almost perfect agreement with the skull deformity diagnosed by standard of reference for both radiologists (Cohen’s κ coefficient = 0.92, 95% CI, 0.77–1.00 for radiologist 1 and κ = 0.76, 95% CI, 0.51–1.00 for radiologist 2). Moreover, rating of the grade of fusion over all calvarial sutures on CT-like images compared to the standard of reference showed a substantial agreement for both radiologists (Cohen’s κ coefficient = 0.76, 95% CI 0.51–1.00 for radiologist 1 and κ = 0.69, 95% CI, 0.41–0.96 for radiologist 2).

**Table 3:** Agreement between the diagnosis based on CT-like imaging and the confirmed diagnosis; interobserver and intraobserver agreement

	Agreement between diagnosis based on CT-like imaging and the confirmed diagnosis		Interobserver and intraobserver agreement	
	Radiologist 1 ^b^	Radiologist 2 ^b^	Interobserver ^c^	Intraobserver ^c^
Main synostosis	0.92 (0.77, 1.00)	0.76 (0.51, 1.00)	0.84 (0.63, 1.00)	1.00 (1.00, 1.00)
Skull deformity	0.92 (0.77, 1.00)	0.76 (0.51, 1.00)	0.84 (0.63, 1.00)	1.00 (1.00, 1.00)
Fracture	1.00 (1.00, 1.00)	1.00 (1.00, 1.00)	1.00 (1.00, 1.00)	1.00 (1.00, 1.00)

^a^Data in brackets are 95% confidence intervals

^b^Agreement between the diagnosis based on CT-like imaging and the confirmed diagnosis using the Cohen’s κ coefficient

^c^Interobserver and intraobserver agreement for diagnosis based on CT-like imaging using the Cohen’s κ coefficient

The sensitivity and specificity for the correct identification of the diagnosis using the MR-derived CT-like images was high, with a sensitivity of 0.94 (95% CI, 0.73, 0.99) for radiologist 1 and 0.88 (95% CI, 0.66, 0.97) for radiologist 2. The specificity was 100% for both radiologists. The subjective diagnostic certainty overall using MR-derived CT-like images was very high, with an average certainty of 87.3 ± 15.4% for radiologist 1 and 82.7 ± 15.1% for radiologist 2. The average overall image quality was rated 1.1 ± 0.3 (radiologist 1) and 1.3 ± 0.5 (radiologist 2) on the abovementioned 4‑point Likert scale. The interrater reliability for assessment of the final diagnosis using MR-derived CT-like images was substantial with κ = 0.84 (95% CI, 0.63, 1.00); the intrarater reliability was κ = 1.00 for both radiologists. Also, interrater reliability for rating the grade of fusion over all calvarial sutures was substantial (Cohen’s κ coefficient = 0.75, 95% CI, 0.49–1.00); the intrarater reliability was κ = 1.00 for both radiologists.

### Patients with Skull Fractures

**Fig. 4:**
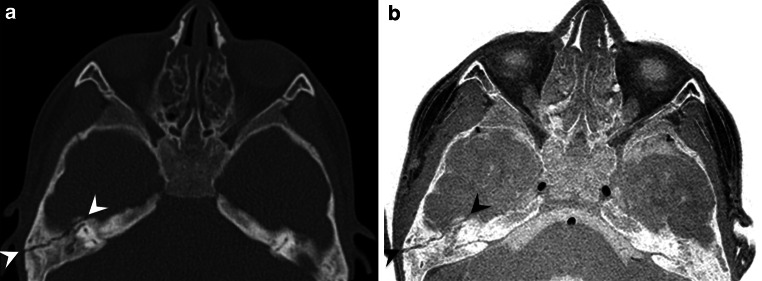
Axial reformation of a CT (**a**) and inverted 3D T1-weighted spoiled gradient echo sequence (**b**) of the skull of a 6-year-old patient with a non-displaced fracture of the right temporal bone (*arrows*)

**Fig. 5:**
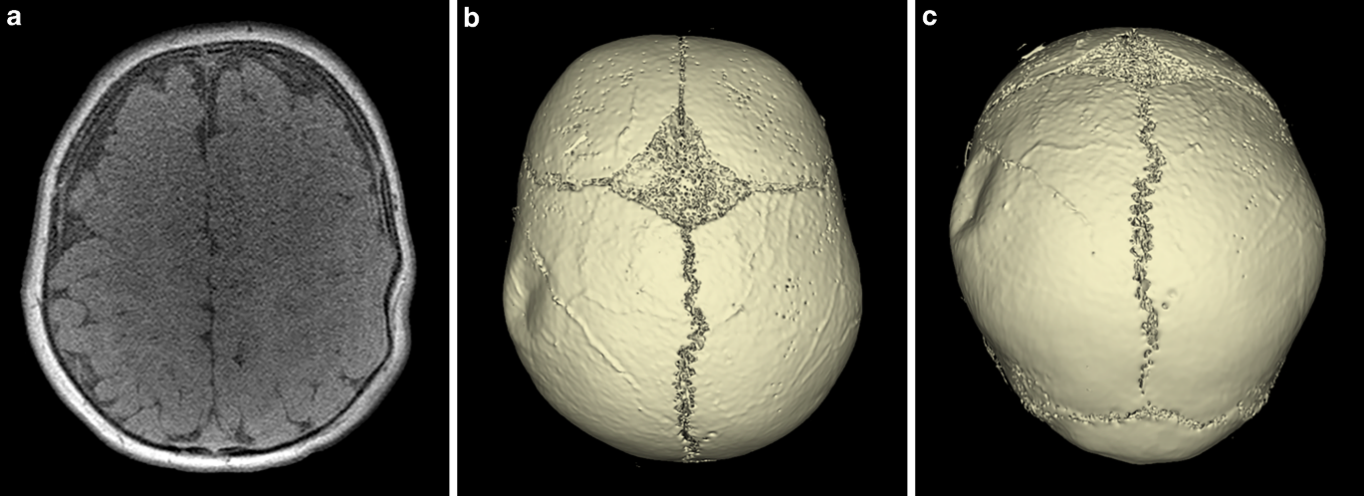
Axially acquired 3D T1-weighted spoiled gradient echo sequence (**a**) with which 3D reconstructions were performed (**b** and **c**) of the skull of a 6-month-old male patient with a ping pong fracture of the left parietal bone. It should be noted that the unfused sutures can clearly be depicted on the 3D reconstructions

Of the two patients with skull fractures, one patient showed a fracture of the right temporal bone (Fig. 4) and one a ping pong skull fracture of the left parietal bone (Fig. 5). For the patients with skull fractures, the diagnosis based on the 2D CT-like images and the 3D reconstructions of the CT-like images showed a perfect agreement with the final diagnosis for both radiologists (Cohen’s κ coefficient = 1.00, 95% CI, 1.00–1.00). The subjective diagnostic certainty for the diagnosis was very high overall, with an average certainty of 100% for radiologist 1 and 90.0% for radiologist 2. For patients with a skull fracture, the average overall image quality was rated 1.5 ± 0.5 (radiologist 1) and 2.0 ± 0.0 (radiologist 2) on the abovementioned 4‑point Likert scale.

## Discussion

In our study we could demonstrate that imaging of the pediatric skull using a high-resolution CT-like T1SGRE MR-sequence is a radiation-free method that enables a detailed analysis and visualization of the calvarial bone structures in patients with craniosynostoses and skull fractures.

While radiography is a simple and accessible method that allows a basic documentation of the skull shape and the sutures, CT imaging with 3D image reconstruction enables an even higher degree of detail for the analysis, especially in complex abnormalities [19, 20]; however, radiation exposure is particularly of concern in children [8, 9]. Ultrasound as a radiation-free option is a sensitive method for the evaluation of skull sutures and fractures [21, 22]. Yet, ultrasound has its limitations, such as the lack of 3D visualization possibilities for optimal preoperative assessment and documentation. Moreover, ultrasound highly depends on the degree of experience of the radiologist or physician performing the examination. Prior to surgical intervention, the intracranial structures are routinely evaluated using MR imaging in most children with craniosynostosis in order to assess intracranial pathologies, such as evidence for elevated intracranial pressure or other cerebral abnormalities [23, 24]. As this examination is already performed, MR imaging for visualization of the skull could reduce radiation exposure significantly.

In previous studies, so-called black bone imaging was used to visualize the skull to diagnose craniosynostosis. BB craniofacial imaging was first described in 2012 by Eley et al. in a study that introduced a gradient echo sequence with short TE, short TR and a flip angle of 5° for imaging, which generates images with low signal in osseous structures. As a result, BB-MR imaging allowed the visualization of the calvarian bone, sutures, and the craniofacial skeleton through enhanced contrast between bone and soft tissue. In a subsequent study the sequence was applied in 17 pediatric patients and it was demonstrated that BB-MR imaging enabled 3D visualization of the skull, sutures, and craniofacial skeleton [10–12]. Saariko et al. compared BB-MR imaging using a T1 VIBE sequence and CT in nine pediatric patients and concluded that BB-MR imaging is a valid alternative to 3D-CT in the preoperative evaluation of patients with craniosynostosis [23]. In a further study, Patel et al. concluded that BB imaging using a GA-VIBE sequence on a 3 T MRI is a promising method to achieve CT-like 3D cranial bone images in 11 pediatric patients [14].

In our study we demonstrated that the T1SGRE sequence may be able to perform the visualization of the skull in a form which is generally expected when applying CT: generating 3D high resolution images of the pediatric skull with a CT-like impression, which can be reconstructed in every plane, for the reliable assessment of pathologies, such as craniosynostosis or fractures without exposing the patient to radiation. The technique not only enables a highly detailed visualization of the skull and its sutures but, as our study demonstrates, is also a method for reliable diagnosis of craniosynostosis: The radiological findings based on the T1SGRE were largely consistent with the final diagnosis assessed intraoperatively and when assessing the other imaging modalities applied.

In comparison to previous studies, the patient number in our study is larger, providing 22 full datasets. Also, the T1SGRE sequence generates images with a particularly high spatial resolution. This enables a highly detailed visualization of the skull while still providing an excellent contrast to the surrounding soft tissue; this is particularly important for the detection of very subtle pathologies, which includes non-dislocated fractures or partially fused sutures. Due to the high resolution of the 3D T1SGRE reformations can easily be performed in every plane, and the CT-like impression can be provided by simply inverting the images on the scanner or with the image viewer, which can easily be performed at the scanner or using any image viewer by the reader. All relevant information about the intracranial structures as well as a highly detailed visualization of the skull is immediately available after image acquisition.

Due to the high resolution of the gradient-echo sequence, it is also possible to generate 3D image reformations; the skull shape and the surface details, including the sutures are visible, similar compared to 3D reformations of CT scans. It has been reported that 3D visualization improves the visibility of cranial sutures notably with results of 90.7–100% in 3D-CT compared to 83.7–95.5% on axial CT images [19, 20]. Therefore, the ability to provide three-dimensional reformations with this technique could also increase the overall diagnostic certainty. Due to the high contrast between the soft tissue and the lamina interna/externa the 3D reformations can be generated by setting a threshold that separates soft tissue from bone.

However, 3D reconstructions, similar to the reconstructions of the BB sequences reported by Eley et al. or Kralik et al. are fairly time consuming as multiple segmentation steps are necessary [12, 25]. Yet, after segmentation and subtraction of the surrounding air, setting a suitable threshold separates the osseous structures from the surrounding soft tissue as well as the intracranial structures. This process usually takes only a few minutes, which contrasts the individual segmentation times of up to 2 h as described by previous studies [14]. This time-consuming manual segmentation was already addressed by automated segmentation techniques, but these techniques have only been applied to BB-MR images of the skull of adults so far [15, 16]. The application of these techniques in pediatric patients might be challenging due to the different composition of the osseous, cartilaginous and soft tissue structures of and around the skull; however, automated segmentation of the skull using the T1SGRE sequence seems achievable due to the excellent contrast to the soft tissue and the intracranial structures and should therefore be explored in future studies.

Another pathology for which this sequence could be used is the detection of fractures. Especially ping pong fractures are seen in newborns because of the soft and resilient nature of their bones, similar to a greenstick fractures of long bones and a fracture line is not visualized radiologically [26], which makes the assessment with modalities such as ultrasound challenging.

A general limitation of this method is the rather long acquisition time of 4 min and 55 s on average. This is particularly problematic in children that do not undergo anesthesia for the examination. Termination of the image acquisition due to excessive motion artifacts is always a possibility. Therefore, general anesthesia is needed in many cases in order to acquire the CT-like images; however, this problem could be approached by utilizing novel AI-based methods, e.g. by using compressed sensing in combination with deep learning-based algorithms as presented in recent literature, which could accelerate image acquisition significantly without the loss of the diagnostic accuracy of the sequence [27]. Another general limitation of the 3D T1SGRE technique is a signal loss that occurs in areas with an air-bone interface. This is particularly problematic as there are air-filled cavities in the craniofacial skeleton like the paranasal sinuses; however, this limitation is negligible as the paranasal sinuses are, for the most part, not developed yet in newborns and infants (i.e. when diagnosing craniosynostosis). Moreover, our patient cohort size was fairly small (especially for skull fractures), therefore, it is very important to note that future studies including more patients are needed for further evaluation of the sequence.

Using CT-like MR-based images, derived from a 3D T1SGRE sequence for diagnosing craniosynostosis or other pathologies of the pediatric skull, such as fractures, was feasible in our study. The sequence can easily be included in the MR examination, which is routinely performed in order to assess intracranial structures prior to surgical intervention. The technique allows a detailed evaluation of sutures and fracture lines due to the high resolution and since this sequence is a 3D MR imaging sequence, reformations of the skull could be acquired in every plane as well as 3D reconstructions of the entire skull can be obtained. Thus, all relevant information of the intracranial structures as well as the skull may be obtained in one single imaging examination without exposing the patient to radiation.
